# A retrospective study of real-world effectiveness and safety of rivaroxaban in patients with non-valvular atrial fibrillation and venous thromboembolism in Saudi Arabia

**DOI:** 10.7717/peerj.13974

**Published:** 2022-09-09

**Authors:** Hind M. Alosaimi, Saeed Alqahtani, Bander Balkhi, Mishari Alqahtani, Faisal Alzamil, Abdulaziz Alhossan, Fatmah S. Alqahtany, Abdullah A. Alharbi, Nawaf Abdullah Alqahtani, Hanan Albackr, Ghada Elgohary, Farjah H. Algahtani

**Affiliations:** 1 Clinical Pharmacy Department, College of Pharmacy, Riyadh Elm University, Riyadh, Saudi Arabia; 2 Pharmacy Services, King Fahad Medical City, Riyadh Second Health Cluster, Riyadh, Saudi Arabia; 3 Department of Clinical Pharmacy, College of Pharmacy, King Saud University, Riyadh, Saudi Arabia; 4 Hematopathology Unit, Department of Pathology, College of Medicine, King Saud University, King Saud University Medical City, Riyadh, Saudi Arabia; 5 College of Medicine, King Saud Bin Abdul Aziz University for Health Sciences, Riyadh, Saudi Arabia; 6 Department of Medicine, College of Medicine, King Saud University Medical City, King Saud University, Riyadh, Saudi Arabia; 7 Department of Adult Hematology/Oncology, King Saud University Medical City, King Saud University, Riyadh, Saudi Arabia; 8 Clinical Hematology and Bone Marrow Transplantation Unit, Division of Internal Medicine, Ain Shams University, Cairo, Egypt; 9 Division of Oncology/Hematology, Department of Medicine, College of Medicine, King Saud University, Kind Saud University Medical City, Riyadh, Saudi Arabia

**Keywords:** Rivaroxaban, Non-valvular atrial fibrillation, Venous thromboembolism, Oral anticoagulants, Deep vein thrombosis

## Abstract

**Background:**

Real-world evidence on factor Xa inhibitor (rivaroxaban) prescribing patterns, safety, and efficacy in patients with non-valvular atrial fibrillation (NVAF) and venous thromboembolism (VTE) is rare. Herein, we sought to examine the above outcomes in the largest academic center in the Kingdom of Saudi Arabia (KSA).

**Methods:**

This is a retrospective observational study designed to examine the prescribing pattern, safety and real-world effectiveness of the factor Xa inhibitor rivaroxaban in patients with NVAF and VTE. Data on rivaroxaban prescriptions were collected and analyzed. Bleeding outcomes were defined as per the International Society on Thrombosis and Hemostasis (ISTH) definition.

**Results:**

A total of 2,316 patients taking rivaroxaban recruited through several departments of King Saud University Medical City (KSUMC). The mean age was 61 years (±17.8) with 55% above the age of 60 and 58% were females. Deep vein thrombosis and pulmonary embolism (VTE) was the most prevalent reason for prescribing rivaroxaban, followed by NVAF. A total daily dosage of 15 mg was given to 23% of the patients. The incidence rate of recurrent thrombosis and recurrent stroke was 0.2%. Furthermore, rivaroxaban had a 0.04 percent incidence rate of myocardial infarction. Half of the patients with recurrent thrombosis and stroke were taking 15 mg per day. The incidence rate of major bleeding was 1.1%. More over half of the patients who experienced significant bleeding were taking rivaroxaban at a dosage of 20 mg per day. According to the HAS-BLED Score (>2 score), 48 percent of patients who experienced significant bleeding had a high risk of bleeding. Non-major bleeding occurred in 0.6% of cases. Similarly, 40% of patients with non-major bleeding were taking rivaroxaban at a dosage of 20 mg per day. According to the HAS-BLED Score, just 6.6% of these individuals had a high risk of bleeding. 93.4% of the patients, on the other hand, were at intermediate risk.

**Conclusion:**

The prescription of rivaroxaban in this real-life cohort study differs from the prescribing label and the outcomes of a phase 3 randomised clinical trial. However, for individuals with VTE and NVAF, the 20 mg dose looked to be more efficacious than the pivotal trial outcomes. Furthermore, among patients with VTE and NVAF, rivaroxaban was linked to a decreased incidence of safety events such as recurrent thrombosis, recurrent stroke, MI, major bleeding, and non-major haemorrhage in a real-world environment.

## Introduction

Deep vein thrombosis (DVT) and pulmonary embolism (PE) are two manifestations of venous thromboembolism (VTE), and they have similar predisposing factors (Torbicki et al., 2008). VTE is the third most frequent cardiovascular disease in the world, and it is one of the main causes of mortality (Goldhaber, Visani & De Rosa, 1999). Anticoagulant therapy is the cornerstone of VTE treatment (Cabral & Ansell, 2015). Warfarin was the only available option as an oral anticoagulant (OAC) for more than 50 years for the treatment of atrial fibrillation and other thrombotic conditions (Julia & James, 2017). Moreover, the use of warfarin has also been linked with a large number of safety reports and associated with several adverse events during routine clinical practice (McMahan et al., 1998; Hylek et al., 2007; Lee & Crowther, 2011).

However, novel oral anticoagulants (NOACs) or direct oral anticoagulants (DOACs) such as rivaroxaban, edoxaban and apixaban are recently approved factor Xa inhibitors that provide anticoagulation *via* oral route (Tellor et al., 2015). These NOACs have expanded the options of OACs available to the healthcare professionals. Due to numerous challenges with warfarin therapy including frequent monitoring, drug interactions, delayed time to onset and a narrow therapeutic index, make the NOACs attractive treatment options (Ageno et al., 2012).

In 2011, rivaroxaban was initially approved by the US. Food and Drug Administration in knee and hip replacement surgery patients for preventing DVT (Janssen Pharmaceuticals Inc, 2018). In the same year, it was approved for the prevention of stroke and systemic embolism in non-valvular atrial fibrillation (NVAF). Whereas in 2012, rivaroxaban was also approved for the treatment of DVT and PE and for the risk reduction of recurrent DVT and PE (Burness & Perry, 2014).

The oral factor Xa inhibitors such as rivaroxaban represent a major advance in the prevention and treatment of thromboembolic disease (Rupprecht & Blank, 2010). Rivaroxaban is potent, oral, highly selective direct inhibitor of factor Xa with high oral bioavailability (Rupprecht & Blank, 2010). It works effectively for DVT and PE therapy, as well as primary and secondary thromboprophylaxis (Rupprecht & Blank, 2010). Direct factor Xa inhibitors inhibit platelet activation and fibrin clot formation *via* direct, selective and reversible inhibition of factor Xa (Rupprecht & Blank, 2010). These agents have several advantages over the vitamin K antagonists such as rapid onset of action, no requirement for dose adjustment, and few drug and food interactions. Thus, these agents become widely used in the treatment and prevention of all VTE related diseases.

Although the efficacy and safety of the factor Xa inhibitor for the prevention of VTE and stroke in patients with NVAF were shown in global clinical trials, the safety and effectiveness data from unselected patients in everyday clinical practice are yet limited. Recently, the use of those medications was increased which raise a concern about it risk-benefit ratio specially in real practice in our region. Real-world data on Factor Xa inhibitor are essential in determining whether evidence from randomized controlled clinical trials translate into meaningful clinical benefits for patients in everyday practice. Overall, only limited number of studies have been published assessing the real-life use of direct oral Factor Xa inhibitors. The aim of this study is to investigate the prescribing pattern, efficacy and safety of Factor Xa inhibitor (rivaroxaban) in real-world clinical practice in the KSA.

## Materials and Methods

### Design and setting

This was a retrospective observational study designed to examine the use of rivaroxaban in patients with documented symptomatic VTE or documented non-valvular atrial fibrillation. Data collected from January 2015 to December 2019 from a large academic center were analyzed.

The primary safety outcome in this study is major bleeding (MB) and clinically relevant nonmajor bleeding (CRNMB). Major bleeding is defined according to International Society on Thrombosis and Hemostasis (ISTH) major bleeding can be defined as any fatal bleeding or bleeding that causes a decrease of hemoglobin level in blood dropped to 20 g/dL or which required a transfusion of packed blood cells of atleast two units, or any hemorrhage into any major antomical sites whether intracranial, peritoneal, pericardial *etc*. Those which does not satisfy criterion for major bleeding constitutes non major bleeding types (Schulman, Kearon & Subcommittee on Control of Anticoagulation of the Scientific and Standardization Committee of the International Society on Thrombosis and Haemostasis, 2005). Patients weighing ≤50 kg and/or aged ≥75 years were analyzed separately.

The primary effectiveness endpoints were recurrent thrombosis which was defined as a new sudden thrombosis confirmed by Duplex ultrasound or CT scan, V/Q scan; recurrent stroke which was defined as a neurological deficit due to any vascular abnormalities persisting beyond a day; noncentral nervous system side effect which was defined as any sudden vascular insufficiency with instances of arterial occlusion other than due to trauma atherosclerosis *etc*.; and myocardial infarction defined as inclusion of myocardial symptoms together with increase in level of cardiac biomarkers (troponin I, troponin T, or creatinine kinase-MB) above normal upper limit.

The secondary outcomes includes all-cause mortality, and rate of treatment discontinuation. All outcomes were stratified by major comorbidities such as stroke or bleeding (*e.g*., CHADS2 or HAS-BLED), other baseline subgroups (*e.g*., age, body weight, CrCl, use of antiplatelet agents, or prior stroke/transient ischemic attack/noncentral nervous system SE), or with different rivaroxaban dosing (10, 15, 20 mg).

### Data collection and ethical approval

The following baseline data was collected: age, sex, body weight, height, smoking history, and any history of allergy, history of VTE, NVAF and other medical history, vital signs and laboratory tests, use of an anticoagulant or antiplatelet agent ≤30 days prior to rivaroxaban administration. Recurrent thrombosis/stroke and bleeding risk profiles based on risk scores such as: D-dimer, thrombophilia, CHADS2 (congestive heart failure, hypertension, age, diabetes mellitus, stroke) or HAS-BLED (hypertension, abnormal liver/renal function, stroke history, bleeding predisposition, labile international normalized ratio, elderly, drug/alcohol use). This study was initiated after the approval of research ethics committee of King Saud University Medical City (ethical approval reference number: E-19-4514) and written informed consent was obtained from the participants.

### Statistical analysis

For baseline characteristics, descriptive analysis was performed and categorical variables are compared using the two-sided Chi-square and Fisher’s Exact tests.

## Results

### Baseline characteristics

A total of 2,316 patients with a mean age of 60.9 years (±17.8) were enrolled in this study. More than half (55.3%) of the patients were over 60 years of age. Fifty-eight percent of the study participants were female. Table 1 shows the baseline and clinical characteristics of the study patients. The average ± SD body weight was 80.3 ± 19.9, height was 1.61 ± 0.09 and the BMI was 30.9 ± 7.4. Two-third of the patients were obese. The prescriptions for VTE (DVT and PE) were relatively higher than NVAF. In a group of VTE, 76% of patients had DVT and 24% were diagnosed with PE.

**Table 1:** Demographics and clinical characteristics of study patients.

Variables	Overall (*n* = 2,316)
Age, years; mean ± SD	60.9 ± 17.8
≤60 years	1,035 (44.7)
>60 years	1,281 (55.3)
Gender; *n* (%)	
Male	963 (41.6)
Female	1,353 (58.4)
Weight, kg; mean ± SD	80.3 ± 19.9
Height, m; mean ± SD	1.61 ± 0.09
BMI, kg/m^2^; mean ± SD	30.9 ± 7.4
Underweight (<18.5)	45 (1.9)
Normal-weight (18.5–22.9)	220 (9.5)
Overweight (23–27.5)	513 (22.2)
Obese (>27.5)	1,538 (66.4)
Smoking Status; *n* (%)	89 (3.8)
VTE Hx; *n* (%)	684 (29.5)
DVT	520 (76)
PE	164 (24)
NVAF Hx; *n* (%)	521 (22.5)
Atrial fibrillation	452 (86.8)
Atrial flutter	23 (4.4)
Unknown atrial rhythm	46 (8.8)
Daily dose of rivaroxaban, *n* (%)	
10 mg	579 (25)
15 mg	537 (23)
20 mg	1,168 (50)
30 mg	32 (2)
Antiplatelets, *n* (%)	
Aspirin; *n* (*n*%)	496 (21.4)
Clopidogrel; *n* (%)	76 (3.3)
Comorbidities; *n* (%)	
Hypertension	1,147 (49.5)
Dyslipidemia	439 (19)
Hypothyroidism	180 (7.8)
Renal dysfunction	132 (5.7)
Heart failure	270 (11.7)
Diabetes mellitus	952 (41.1)
ALT, unit/L; mean ± SD	35 (67.9)
ALT groups; *n* (*n*%)	
≤41 units/L	2,040 (88.1)
42–123 units/L	236 (10.2)
>123 units/L	40 (1.7)
AST, unit/L; mean ± SD	30.6 (148)
AST groups; *n* (*n*%)	
≤31 units/L	2,015 (87)
32–93 units/L	248 (10.7)
>93 units/L	53 (2.3)
ALP, unit/L; mean ± SD	98.9 (72.3)
ALP groups; *n* (*n*%)	
≤150 units/L	2,126 (91.8)
151–450 units/L	179 (7.7)
>450 units/L	11 (0.5)
Albumin, gm/L; mean ± SD	34.6 (56.2)
Albumin groups; *n* (*n*%)	
≤50 g/L	2,308 (99.7)
>50 g/L	8 (0.3)
Bilirubin, mcmol/L; mean ± SD	12.4 (19.4)
Bilirubin groups; *n* (*n*%)	
≤17 mcmol/L	2,062 (89)
>17 mcmol/L	254 (11)
Serum creatinine; mcmol/L; mean ± SD	84.4 (51)
Serum creatinine groups; *n* (*n*%)	
≤106 mcmol/L	1,969 (85)
>106 mcmol/L	347 (15)
Creatinine clearance; ml/min; mean ± SD	111.4 (61.7)
Creatinine clearance groups; *n* (*n*%)	
≤M:137/F:128 ml/min	1,613 (69.6)
>M:137/F:128 ml/min	703 (30.4)

**Note:**

BMI, body mass index; NVAF, nonvalvular atrial fibrillation; DVT, deep venous thrombosis; PE, pulmonary embolism; ALT, alanine aminotransferase; AST, aspartate aminotransferase; ALP, alkaline phosphatase, ALT: 7–55 units per litre (U/L); AST: 8–48 units per litre (U/L); ALP: 44–147 units per litre (U/L); albumin: 3.5–5.5 grams per decilitre; bilirubin(total): 0.3–1.0 milligrams per decilitre; indirect bilirubin: 0.2–0.8 milligrams per decilitre. Normal serum creatinine levels: For adult men: 0.74–1.35 milligrams per decilitre (Mg/dL); for adult women: 0.59–1.04 milligrams per decilitre (Mg/dL).

### Rivaroxaban dosing

We observed four different total daily dosing of rivaroxaban 10, 15, 20, and 30 mg. The most common dose on these patients was 20 mg/day with 50% of the patients using this dose. While the 10 mg dose was prescribed in 25% of patients followed by 15 mg for 23% of the patients. A total of 30 mg dose were only used by 2% of the patients.

### Antiplatelets therapy and comorbidities

Concomitant use of antiplatelets was noted in 572 patients (24%) The prevalence of comorbidities ranged from 5.7% to 49.5%, with hypertension being diagnosed in 49.5% of our cohort. Diabetes and dyslipidemia were reported in 41.1%, 19%, respectively (Table 1). Prevalence rates for renal dysfunction, hypothyroidism, and heart failure ranged from 5.7%, 7.8% and 11.7%.

### CHADS2 score and HAS-BLED score

CHADS2 Score and HAS-BLED Score were calculated for the patients and presented in Table 2. Nearly half of the patients had an intermediate risk of stroke per CHADS2-Score. A total of 437 patients (18.9%) had a high risk of stroke and 31.5% of patients had a low risk of stroke. On the other hand, majority of the patients (61.2%) had an intermediate risk of bleeding as per HAS-BLED score. 26.9% of the patients had a low risk for bleeding and 11.9% had high risk for bleeding.

**Table 2:** Risk of stroke and major bleeding in study patients on different dosing.

Variables		Total daily dose of rivaroxaban				
	Overall (*n* = 2,316)	10 mg (*n* = 579)	15 mg (*n* = 537)	20 mg (*n* = 1,168)	30 mg (*n* = 32)	*P*-value
Risk of stroke as per CHADS_2_-score; *n* (%)						
Low risk	730 (31.5)	215 (37.1)	87 (16.2)	416 (35.6)	12 (37.5)	0.892
Intermediate risk	1,149 (49.6)	296 (51.1)	284 (52.9)	556 (47.6)	13 (40.6)	0.062
High risk	437 (18.9)	68 (11.7)	166 (30.9)	196 (16.8)	7 (21.9)	0.537
Risk of major bleeding as per HAS-BLED score; *n* (%)						
Low risk	622 (26.9)	187 (32.3)	64 (11.9)	362 (31)	9 (28.1)	0.834
Intermediate risk	1,418 (61.2)	349 (60.3)	370 (68.9)	677 (58)	22 (68.8)	0.172
High risk	276 (11.9)	43 (7.4)	103 (19.2)	129 (11)	1 (3.1)	0.505

### Effectiveness outcomes

Effectiveness outcome variables listed in Table 3 indicate the following parameters. The table evaluated recurrent thrombotic and stroke events. Those having 10 and 30 mg rivaroxaban dose showed nil for the study and cases having 15 and 30 mg dosages each attributed to 50% recurrent thrombotic and stroke incidences. While the total incidence rate measured 0.2% for each of the variables, it summed up to 0.04% for MI. There was no association between the recurrent rates and the dosage of rivaroxaban (Table 3). All of the patients who had recurrent thrombosis, stroke and MI had a high risk for VTE according to CHADS2 Score (Table 4). Moreover, there were no association between the rates of recurrent and other variables.

**Table 3:** Effectiveness and safety outcomes of patients.

Variables		Dose of rivaroxaban				
	Overall (*n* = 2,316)	10 mg (*n* = 579)	15 mg (*n* = 537)	20 mg (*n* = 1,168)	30 mg (*n* = 32)	*P*-value
**Effectiveness outcomes**						
Recurrent thrombosis; *n* (%)	4 (0.2)	0	2 (0.4)	2 (0.2)	0	0.513
Recurrent stroke; *n* (%)	4 (0.2)	0	2 (0.4)	2 (0.2)	0	0.513
Myocardial infarction; *n* (%)	1 (0.04)	0	0	1 (0.1)	0	0.805
**Safety outcomes**						
Major bleeding; *n* (%)	25 (1.1)	4 (0.7)	6 (1.1)	15 (1.3)	0	0.652
Minor bleeding; *n* (%)	15 (0.6)	4 (0.7)	5 (0.9)	6 (0.5)	0	0.748

**Note:**

*P* > 0.05 Not significant.

**Table 4:** Effectiveness and safety outcomes of patients.

Variables		Risk of stroke according to CHADS2-score			
	Overall (*n* = 2,316)	Low risk (*n* = 730)	Intermediate risk (*n* = 1,149)	High risk (*n* = 437)	*P*-value
**Effectiveness outcomes**					
Recurrent thrombosis; *n* (%)	4 (0.2)	0	0	4 (0.9)	0.732
Recurrent stroke; *n* (%)	4 (0.2)	0	0	4 (0.9)	0.861
Myocardial infarction; *n* (%)	1 (0.04)	0	0	1 (0.2)	0.236
		**Risk of bleeding according HAS-BLED score**			
		**Low risk (*n* = 622)**	**Intermediate risk (*n* = 1,418)**	**High risk (*n* = 276)**	
**Safety outcomes**					
Major bleeding; *n* (%)	25 (1.1)	0	13 (0.92)	12 (4.3)	0.141
Minor bleeding; *n* (%)	15 (0.6)	0	14 (0.98)	1 (0.35)	0.516

### Safety outcomes

Major bleeding episodes were documented in 25 patients (1.1%) (Table 3). Approximately half of those patients who had major bleeding were on 20 mg daily dose of rivaroxaban. A total of 48% of the patients who had major bleeding had a high risk for bleeding according to HAS-BLED Score (>2 score) (Table 4). No patients with low risk HAS-BLED score had an event of major bleeding.

Fifteen patients had clinically relevant non-major bleeding (0.6%). 40% of the patients who had non-major bleeding were on 20 mg daily dose of rivaroxaban. Only 6.6% of these patients had a high risk for bleeding according to HAS-BLED Score (Table 4). 93.4% of the patients had moderate risk (Table 4). Moreover, there were no association between the rates of bleeding and other variables.

## Discussion

Although, the randomized control trials (RCTs) remain the gold standard in assessing drugs safety and efficacy, these studies do not, necessarily, reflect the real clinical practice. To our knowledge, this is the first study in the Kingdom of Saudi Arabia to assess the prescribing pattern, effectiveness and safety of rivaroxaban in patients with VTE and or NVAF in a large cohort. The findings of this study are based on a real-world clinical practice’s data collected from a tertiary care healthcare setting during a period of 5 years.

This study revealed several important findings about the effectiveness and the incidence of safety events. Interestingly, the prescriptions of rivaroxaban for females particularly with VTE were relatively higher than their male counterparts. However, the role of gender in the causation of VTE is uncertain (Tormene et al., 2011). A large number of study participants were aged 60 years and over which is consistent with previous VTE and NVAF literature study as the incidence of VTE and NVAF increases with age (ESHRE Capri Workshop Group et al., 2013). Patients in our study mostly administered rivaroxaban for the treatment of VTE.

In this study we noted that half of the patients received appropriately a 20 mg total daily dose of rivaroxaban for VTE. On the other hand, 23% of patients were taking 15 mg total daily dose. For the subgroups of VTE such as DVT and PE, the recommended dose of rivaroxaban is 15 mg orally twice a day followed by 20 mg once daily and the treatment continues for as long as the risk of VTE recurrence persists, balancing benefits and harms and patient preference (Streiff et al., 2016). This unusual dosing was also noted in the RIvaroxaban for Valvular heart diseasE and atRial fibrillation (RIVER) study were 76.5% of the patient received rivaroxaban 20 mg and 20% received 15 mg while 10 mg dose was prescribed in only 2.1% (Beyer-Westendorf et al., 2019).

Regarding the effectiveness outcomes, there was no correlation between the dose and response. In general, the rate of recurrent thrombosis, stroke, and MI was very low in our reported findings. Furthermore, the recurrent rates were lower than what have been reported in the clinical trials (Hori et al., 2014) and other real-world outcomes studies (Patel et al., 2011; Hori et al., 2012, 2014; Laliberté et al., 2014; Tellor et al., 2015; Ntaios et al., 2017; Huang et al., 2018). Moreover, all those patients who had recurrent events had high risk for stroke according to CHADS2 scores. This may suggest using higher doses of rivaroxaban in this group of patients. In general, patients in the present study had less baseline risk for thromboembolism as indicated by lower average CHADS2 scores compared to the previous studies which may explain the more prominent effectiveness of rivaroxaban in our data. The majority of cohort in the current study had an intermediate risk of stroke (49.6) compared to average CHADS2 scores of 3.27 and 3.2 in J-ROCKET AF, ROCKET AF (rivaroxaban once daily oral direct Factor Xa inhibition compared with vitamin K antagonism for prevention of stroke and embolism trial in atrial fibrillation), respectively (Patel et al., 2011; Hori et al., 2012).

Recurrent thrombosis and recurrent stroke were almost nil for those taking rivaroxaban doses of 10 and 30 mg. Myocardial infarction recurrency lined up the same results. On accountable of BMI status, about 66% of cases counted obese, the overweight group calculated to 22%, the underweight percentage being meagre. About 21% were taking aspirin supplements as well. The coupling effect might reduce the risk of secondary athero thrombotic events during immediate post-acute coronary syndrome period. Almost 3% of cases were taking antiplatelet drug clopidogrel for caution of bleeding events.

NVAF was the second most common diagnosis in this study for which patients received the treatment of rivaroxaban with different doses. ROCKET-AF was the first trial assessing the effectiveness of rivaroxaban with a dose of 20 mg in NVAF patients (Patel et al., 2011). ROCKET-AF concluded that rivaroxaban caused less fatal bleeding and was noninferior to warfarin in patients with atrial fibrillation for preventing stroke or systemic embolism. Rivaroxaban has been shown to be well tolerated with superior safety with regards to bleeding events compared with warfarin (Vimalesvaran, Dockrill & Gorog, 2018). Our findings in this study also showed the effectiveness of rivaroxaban in the preventing of stroke or systemic embolism and in agreement with the previous reports.

Rivaroxaban, a NOAC (noval oral anticoagulant) dosage tablets comes under 10, 15 and 20 and 30 mg strengths (Konicki et al., 2020). Dosages were regulated in some cases having other antiplatelet prophylaxis and in those taking vitamin k antagonistics. Dosages were minimised at times in mild contra-indicative cases and even discontinued as precaution for any preoperative surgical criterion. However, the study including the secondary outcomes, the efficacy of 20 mg dose proved better (Blin et al., 2019).

Regarding the safety outcomes, we observed a significantly lower risk of major and non-major bleeding compared to previous studies (Patel et al., 2011; Hori et al., 2012, 2014; Laliberté et al., 2014; Ntaios et al., 2017; Huang et al., 2018). Our study also reported a lower incidence of bleeding events as major bleeding occurred in only 15 patients who received 20 mg dose of rivaroxaban. We noticed that the rate of bleeding increases with the dose as there were few cases in 10 mg dose compared to 15 and 20 mg doses. In addition, we found an association between increase risk of bleeding and occurrence of major bleeding. According to HAS-BLED score, most of the patients who had major bleeding has high and intermediate risk for bleeding. Whereas, no major bleeding events were found in patients with low risk for bleeding. The estimation of bleeding risk through HAS-BLED score is crucial as it helps clinicians to flag-up patients at potential risk for serious bleeding in an informed manner. HAS-BLED score also helps healthcare professionals to explore the potentially reversible risk factors of bleeding. Moreover, the use of CHADS2-score and HAS-BLED scores can help to inform the choice of antithrombotic agent and the management strategy, thus reducing the likelihood of adverse events (Lane & Lip, 2012).

There were some limitations to this study. First, the retrospective design of the study did not allow to control some confounders that may affect the final outcomes of this study. Second, some data were missing as the care of many women was partitioned between different disciplines and, frequently among several hospitals. Third, as it was conducted at a single tertiary care institution in the KSA, the results cannot be generalized to the entire population of the KSA. Fourth, this study had no comparative arm so it is impossible to directly compare the outcomes of rivaroxaban in this study with other NOACs. Therefore, these findings should be validated by a multicenter longitudinal study across tertiary care settings in the KSA.

## Conclusions

In this real-life cohort study, rivaroxaban prescribing is not consistent with the prescribing label or phase 3 randomized clinical trial data. However, it appears that the efficacy of the 20 mg dose is better than pivotal trial data for patients with VTE and NVAF. Moreover, in real world setting, rivaroxaban was associated with a lower risk of safety events such as recurrent thrombosis, recurrent stroke, MI, major bleeding, and non-major bleeding in patients with VTE and NVAF.

In this real-life cohort study, rivaroxaban prescribing for VTE and NVAF is not consistent with evidence of randomized clinical trials. However, the efficiency and safety of this drug is not too different from pivotal phase 3 trials. Subsequent larger scale studies are required to validate these findings.

## Supplemental Information

10.7717/peerj.13974/supp-1 Supplemental Information 1 Raw Data.

## ABBREVIATION

NVAF: Non-valvular atrial fibrillation
VTE: Venous thromboembolism
ISTH: International Society on Thrombosis and Haemostasis
DVT: Deep vein thrombosis
PE: Pulmonary embolism
MB: Major bleeding
CRNMB: Nonmajor bleeding
RCT: Randomized control trials
MI: Myocardial infarction

## Additional Information and Declarations

### Data Availability

The following information was supplied regarding data availability: The raw data is available in the Supplemental File.
